# Spatial disparities of population aging in Shenzhen: from China’s Hukou perspective

**DOI:** 10.3389/fpubh.2025.1614007

**Published:** 2025-08-04

**Authors:** Qing Luo, Zhuoying Li, Yuanjun Li, Yunhao Yang, Jiaxin Liu, Guangbo Liu, Huayi Wu

**Affiliations:** ^1^School of Mathematics and Physics, Wuhan Institute of Technology, Wuhan, Hubei, China; ^2^School of Mathematics and Statistics, Lanzhou University, Lanzhou, Gansu, China; ^3^Guangzhou Institute of Geography, Guangdong Academy of Sciences, Guangzhou, Guangdong, China; ^4^State Key Laboratory of Information Engineering in Surveying, Mapping and Remote Sensing, Wuhan University, Wuhan, Hubei, China

**Keywords:** population aging, China’s Hukou system, immigrant megacity, spatial autocorrelation, spatial Durbin model, sustainable aging polices

## Abstract

**Objective:**

As China’s youngest city with immigrants constituting 66% of its population, Shenzhen is paradoxically facing accelerated and compressed demographic aging. Although its overall aging rate remains below the international aging society threshold, the city confronts unique challenges as a migrant receiving metropolis without domestic precedents. Through a novel perspective of the Hukou system (i.e., the household registration system), this research reveals distinct spatial patterns of the older populations with different Hukou types. The findings aim to inform policy responses to Shenzhen’s upcoming concentrated aging transitions.

**Methods:**

This study integrates administrative-level statistical data with nighttime light data, spatially disaggregated to a 1*1 km grid resolution. Employing spatial autocorrelation analysis, we identify spatial patterns of older populations. The spatial Durbin model is further applied to quantify both direct and spatial spillover effects of economic, social welfare, and natural environmental factors on the two aging cohorts.

**Results:**

Local Hukou holders show a “west to east rising” aging trend, while older population without Shenzhen’s Hukou exhibit a “south high, north low” pattern. Both groups demonstrate strong positive spatial spillover effects within their respective Hukou type. Key influencing factors differ between the groups, with local Hukou older adults prioritizing environmental quality, while non-local Hukou older adults are more closely linked to economic indicators.

**Conclusion:**

Distinct spatial patterns emerge between Hukou-registered and migrant older populations, with three influence categories exhibiting divergent mechanisms across these Hukou-defined cohorts. These dual disparities necessitate tailored policy interventions addressing institutionalized aging inequalities in Shenzhen, and also offering insights for other rapidly urbanizing cities with similar demographic structures.

## 1 Introduction

Population aging is a global issue and one of the most pressing challenges in modern society. According to the UN World Population Prospects, the number of people over 65 will exceed 1.5 billion by 2050 (1). In China, the 7th National Census indicates that by 2020, over 18.7% of the population was aged 60 and above, and more than 13.5% were over 65. These figures surpass the international aging thresholds of 10 and 7%, respectively, signifying that China has entered an aging era. As rapid urbanization continues, it is essential to study population aging within this demographic shift. Additionally, disparities in old-age welfare and resource allocation between local and non-local Hukou older populations contribute to imbalances in aging development.

China’s Hukou system or the household registration system, a key state administrative tool, has evolved from its origins in the Shang Dynasty of ancient China as a basic population registration system to a vital mechanism for tax collection, population management, and law enforcement (2). After the People’s Republic of China was established, the system was further developed to focus on population registration and management. As China shifted from a planned to a market economy, local adaptations led to diverse Hukou models reflecting varying social and economic conditions. Despite national laws like the Regulations on Household Registration, implementation varies due to the country’s vast size and uneven development, affecting policies on migration, social security, welfare, education, and real estate (3). In developed areas, local Hukou residents enjoy priority access to resources, whereas non-locals face restrictions. These disparities increase social inequality, yet areas with more job opportunities continue to draw many immigrants despite these challenges.

Shenzhen, China’s first special economic zone and a pioneer of reform and opening-up, has experienced rapid economic growth. It is the most economically dynamic and open city in the country, with the highest proportion of immigrants. From 2000 to 2020, Shenzhen’s population grew from 7,012,400 to 17,633,800^1^, with non-domiciled residents accounting for approximately 63.36% of this growth. Shenzhen has thus emerged as a new immigrant city with the highest proportion of immigrants nationwide. Known as China’s youngest city, Shenzhen’s youthful demographic is largely due to its immigrant population. Many young people migrate to Shenzhen for entrepreneurial opportunities, delaying the city’s aging process. However, along with the influx of young migrants, some older individuals also relocate to Shenzhen, often to join their children, but without acquiring local Hukou of Shenzhen, leading the older population divided into local and large number of non-local Hukou holders. Although the predominance of young residents minimizes the immediate impact of non-local older adults on aging, over time, they will alter Shenzhen’s age structure. Furthermore, differences in Hukou status can lead to disparities in accessing to public services such as social security, healthcare, and elder care (4, 5), potentially causing imbalanced development between local and non-local aging populations.

Additionally, the pioneering migrants who arrived in Shenzhen during the early stage of its reform and opening-up policy (post-1980) are now entering old age. This cohort, combined with older adults accompanying younger migrants, has led to “compressed aging” in the city. Consequently, Shenzhen’s population aging pattern differs significantly from the gradual transitions observed in other major cities.

While population aging has been extensively studied in urban contexts, the unique dynamics of immigrant cities with substantial migrant populations remain underexplored from China’s Hukou perspective. A common type of existing researches predominantly adopts spatial–temporal analysis frameworks to examine aging patterns. For instance, Chen et al. investigated regional disparities in China’s aging population distribution (6), while Li et al. identified distinct spatial evolution characteristics of aging population in Wuhan, China (7). These studies collectively demonstrate the value of spatial analytics in revealing geographical disparities of aging trends, providing valuable insights for regional policy formulation in care resource allocation for older residents.

Another common type of aging related research focuses on identifying determinants of population aging through multivariate modeling approaches. Researchers typically employ spatial econometric models incorporating socioeconomic, demographic, healthcare, and environmental indicators to disentangle complex influencing mechanisms. Prevailing explanatory frameworks emphasize some dimensions: economic development level, demographic dynamics, educational attainment, urbanization intensity, and environmental quality indices (8–10). For instance, Wang (10) detected spatial spillover positive impacts of social economic factors on population aging of China from a global perspective, Wu and Song (11) concluded that the spatial evolution of China’s population aging was impacted both by demographic factors and economic factors.

The growing accessibility of multi-source geospatial data has revolutionized population studies through advanced spatialization techniques. Because socio-economic data statistics usually take administrative districts as basic study units, which are usually coarse to reflect the spatial heterogeneity within the units, many researchers have employed auxiliary data to gain more informative datasets for the research areas. Xue et al. analyzed the spatial structure of cities and their industry composition mechanism by using the big data on the industry classification of points of interest in 36 cities in three northeastern provinces, China (12). Hao et al. integrated nighttime lighting (NTL) data and population data through carbon emission statistics at the provincial scale, assigning carbon emissions to the raster scale to analyze the spatial and temporal patterns, evolution characteristics, and the correlation between carbon emissions and economic growth in China (13). Zhang et al. proposed a method based on cell phone signaling data to identify people in small activity spaces and their spatial distribution (14).

While extensive research exists on China’s aging population, the institutional duality between Hukou-registered residents and non-registered migrants in major immigrant-receiving cities remains underexplored. This study aims to bridge this gap by investigating the spatial divergence of aging patterns among these two distinct demographic cohorts in Shenzhen – China’s youngest megacity experiencing paradoxical rapid aging. To achieve this end, the study collected the data from the seventh (2020) population census of Shenzhen and NTL data of Shenzhen in 2020, and employed spatial autocorrelation analysis and spatial Durbin model to do the data analysis. The interested variables in this paper are the proportions aged over 65 years of the local Hukou holders and non-local Hukou holders. We also collected economic, social welfare, and environmental factors to reveal their impacting mechanism on population aging of the two groups. In order to gain statistically reliable results, the raw population data were transformed into 1*1 km raster data, and the weights of each raster were calculated using National Polarorbiting Operational Environmental Satellite System Preparatory Project-Visible infrared Imaging Radiometert (NPP-VIIRS) night light data to establish a new dataset of economic, social welfare, and environmental indicators.

The main contributions of this research are as follows: (1) furnishing a novel perspective (the Hukou system) to understand population aging in immigrant cities; (2) uncovering distinct global and local spatial patterns of aging among local Shenzhen Hukou holders and non-local Shenzhen Hukou holders; and (3) highlighting different (spatial spillover) mechanisms of economic, social welfare, and environmental factors influencing these two groups. Notably, the findings reveal that older residents with local Shenzhen Hukou prioritize environmental quality, while those with non-local Shenzhen Hukou are more closely associated with economic indicators. Recommendations are also given from three aspects: healthcare resource allocation, psycho-social infrastructure, and Hukou policy.

The structure of this paper is organized as follows. Section 2 introduces the datasets and methodologies. Section 3 presents the spatial distribution and patterns of population aging in 2020 for both of the two Hukou types. Section 4 discusses the spatial spillover effects of population aging of the two types, as well as the spatial spillover effects of the influential factors on them. Section 5 examines the effects of three types of factors on demographic elements that directly influence aging and offers recommendations for reducing the spatial disparity in population aging between the two Hukou types based on the findings. Finally, section 6 summarizes the results and implications of this work.

## 2 Materials and methods

### 2.1 Data sources and variables

**Figure 1:**
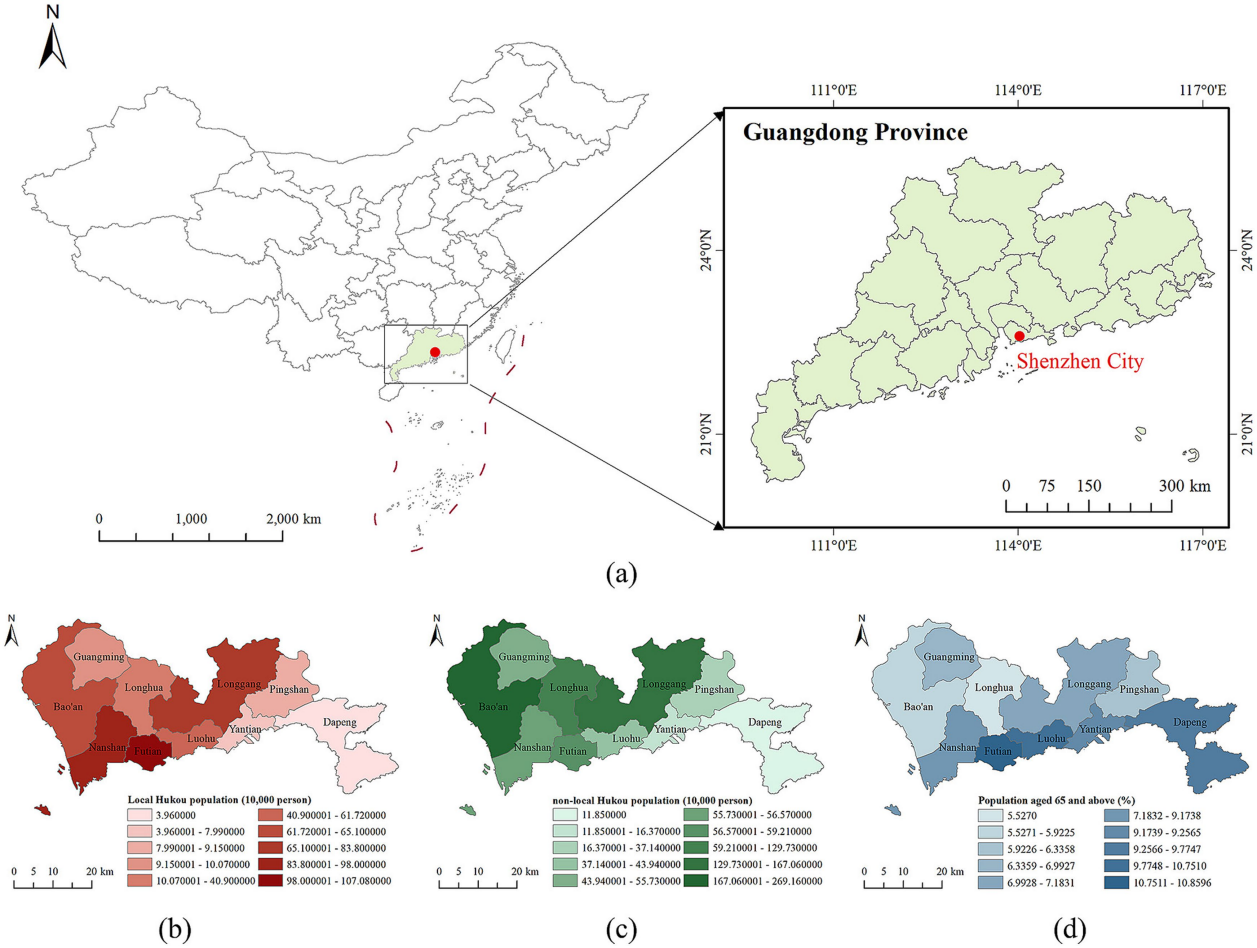
Overview of Shenzhen. **(a)** The geographical location of Shenzhen in China; **(b)** Spatial distribution of permanent residents with Shenzhen Hukou (i.e., local Hukou population); **(c)** Spatial distribution without Shenzhen Hukou (i.e., non-local Hukou population); and **(d)** Spatial distribution of aging rates of 65 years older and above.

The study area of this research is Shenzhen which is a typical immigrant city in China, and the demographical data used in this paper were obtained from the 7th (2020) National Population Census of the National Bureau of Statistics of China. Figure 1 shows geographical location of Shenzhen in China and spatial distributions of some related demographical quantities. The social and economic data and other influencing factors employed in the study were collected from the Statistical Yearbook of Shenzhen and the Statistical Yearbook of Shenzhen districts in 2020. We have two population aging indexes (local Hukou holders aging rate and non-local Hukou holders aging rate) and seven explanatory variables categorized into three types: economic, social welfare, and environmental factors. We also used nighttime light data from NPP-VIIRS to do the spatialization of the population and influential factors data to obtain a larger sample size, ensuring that the statistical analysis can yield reliable results.

#### 2.1.1 Population aging index

In this paper, we used two older population rates (aging rates of 65 years or older of local and non-local Hukou holders) to analyze and discuss aging disparity in Shenzhen. By examining these two rates separately, we aim to gain a more comprehensive understanding of the differences in population aging between the local Hukou and non-local Hukou populations in the city.

#### 2.1.2 Influencing factors

##### 2.1.2.1 Economic indicators

China’s economic development and population aging exhibits obvious spatial correlations (8, 15). This paper employed three economic indicators: the gross domestic product (GDP), gross domestic product growth rate (GDPGR), and gross output value of industries above the designated size (GVA) of Shenzhen districts.

##### 2.1.2.2 Social welfare indicators

We collected data on the number of medical institutions (MI), the urban registered unemployment rate (UUR), and the number of basic pension insurance participants (PIP) as indicators of social welfare (16). The number of MIs reflects the accessibility and capacity of healthcare services in a region. Generally, a higher number of medical institutions suggests better medical care, which is essential for addressing the challenges associated with an aging population (9, 17, 18). The UUR is defined as the proportion of unemployed individuals registered with the State Labor and Social Security Department during the reporting period, relative to the total number of job seekers and unemployed individuals recorded at the end of the period. This rate measures the level of unemployment in a region. Basic pension insurance is a critical part of the social security system; a higher number of participants indicates greater involvement and commitment from both individuals and the government in tackling pension-related issues.

##### 2.1.2.3 Environment indicators

The good air quality rate (GAR) was used as the environmental indicator (19, 20); it is the ratio between the number of days with good air quality and the total number of days monitored in a year. This indicator reflects the local air quality, with higher values indicating better air quality in the area.

In our exploration of the factors influencing population aging, we used the older population rate as the dependent variable and economic, social welfare, and environmental indicators as explanatory variables. All variables are listed in Table 1. To better meet the assumptions of normality, we transformed the raw data for GDP, GVA, PIP, and MI into their natural logarithmic forms.

**Table 1:** Influencing factors of population aging.

Factor category	Indicators	Form in model	Measure
Economic	Gross domestic product (GDP)	ln(GDP)	10 million yuan
	Gross domestic product growth rate (GDPGR)	GDPGR	Billion yuan
	Gross output value of industries above designed size (GVA)	ln(GVA)	–
Social welfare	Urban registered unemployment rate (UUR)	UUR	–
	Number of pension insurance participants (PIP)	ln(PIP)	10,000 people
	Number of medical institutions (MI)	ln(MI)	pc
Environmental	Good air quality rate (GAR)	GAR	–

### 2.2 Data processing

Given that the original sample size of ten is too small for regression analysis, which typically requires larger samples for hypothesis testing, we rasterized the data using nighttime light data as an auxiliary variable. This method is well-established in the literature (21–23). The NTL data utilized in this study are the annual synthetic datasets from the NPP-VIIRS for 2020, corrected using China’s Development of the Long Time Series Nighttime Light Dataset (24). We downloaded the NPP-VIIRS nighttime light data for 2020 to calculate the weight of each grid cell, constructing spatial grid data at a resolution of 1 × 1 km for both dependent and independent variables. This process increased the sample size to 2183.

**Figure 2:**
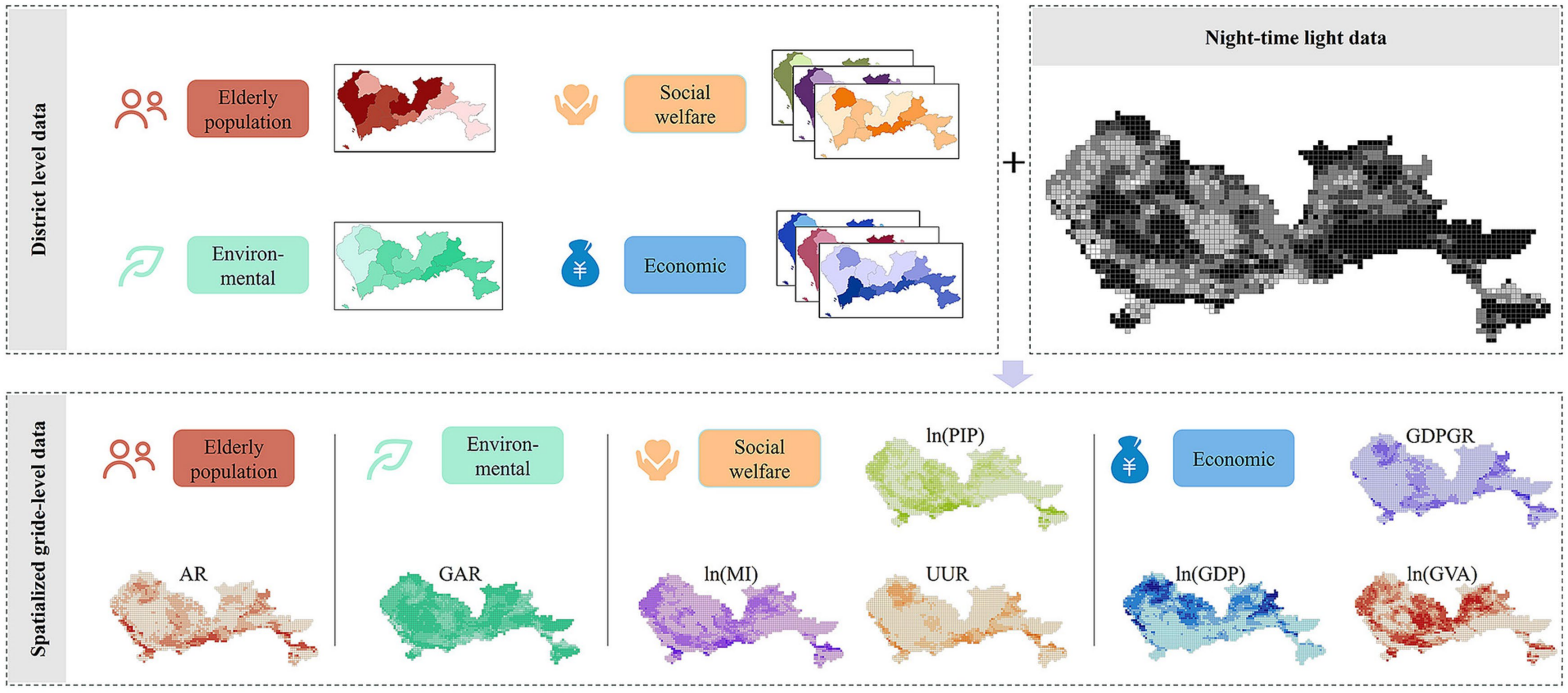
The spatialized population aging index and its influencing factors.

Numerous population spatialization methods have been documented in the literature (25–27). Given the positive correlation between population density and NTL brightness (28, 29), we used the nighttime light index of each pixel as the weight for that grid cell. First, we calculated the total nighttime light index values for each district. Next, we divided the nighttime light index value of each grid by the district’s total to determine the grid’s weight. This process was repeated for all 1 km × 1 km grids. A higher nighttime light index indicates greater brightness on the nighttime lighting map, which correlates with higher GDP (30). Typically, regions with better economic development have higher GDP, along with more developed social welfare and infrastructure. Therefore, we used nighttime light data weights as proxies for economic and social welfare variables. Additionally, researches (31, 32) have shown that air pollution increases with rapid economic development. Consequently, we used the negative value of the nighttime light data weights as proxies for environmental variables. Finally, multiplying these weights by the original values of the collected variables produced rasterized datasets for the independent variables. The procedure of data processing is depicted in Figure 2.

### 2.3 Methods

#### 2.3.1 Global and local autocorrelation analysis

In this study, the Moran’s I (33) was used to quantify the degree of global spatial autocorrelation, while the local indicator of spatial association (LISA) (34), local Moran’s I, was used to measure the degree of local spatial clustering. The Moran’s I indicates whether there is clustering of population aging in the entire Shenzhen area, whereas LISA identifies local clusters. The formula for global Moran’s I is Equation (1):


(1) $$I=\frac{n\sum_{i=1}^{n}\sum_{j=1}^{n}c_{\mathrm{ij}}(x_{i}-\bar{x})(x_{j}-\bar{x})}{\left(\sum_{i=1}^{n}\sum_{j=1}^{n}c_{\mathrm{ij}})(\sum_{i=1}^{n}{\left(x_{i}-\bar{x}\right)}^{2}\right)},$$

here n represents the number of districts in Shenzhen, and $c_{\mathrm{ij}}$ is the entry of the i th row and j th column of the spatial weight matrix C , where $c_{\mathrm{ij}}=1$ if cell i and cell j are adjacent, and $c_{\mathrm{ij}}=0$ otherwise. The variable $x_{i}$ denotes the older population rate in the i th district, and $\bar{x}=\sum_{i=1}^{n}x_{i}/n$ is the mean older population rate. The value of global Moran’s I typically ranges from −1 to 1. A positive global Moran’s I indicates that regions with similar values are clustered together, while a negative value suggests clustering of dissimilar values. A value near zero indicates no spatial autocorrelation, implying spatial randomness. The local indicator of spatial association (LISA) based on Moran’s I is expressed as Equation (2), $I_{i}$ is the Moran’s I value of the i th unit of the study area.


(2) $$I_{i}=(x_{i}-\bar{x})\sum_{j=1}^{n}c_{\mathrm{ij}}(x_{j}-\bar{x}),i=1,2,\cdots ,n$$

#### 2.3.2 Spatial Durbin model (SDM)

The SDM (35) accounts for the spatial autocorrelation of both dependent and explanatory variables. It incorporates spatial lag terms for both the dependent variable and the independent variables. The model is expressed as:


(3) $$Y=\mathrm{\rho WY}+\mathrm{X\beta}+\mathrm{WX\theta}+\varepsilon ,\varepsilon \sim \mathrm{MVN}(0,\sigma^{2}I)$$

In Equation (3), ρ is the spatial autocorrelation coefficient, and W is the row standardized version of the binary spatial weight matrix C , WY is the lag term of the dependent variable Y , and WX is the lag term of the independent variables. β and θ are regression coefficients, while ε denotes the random error term.

**Figure 3:**
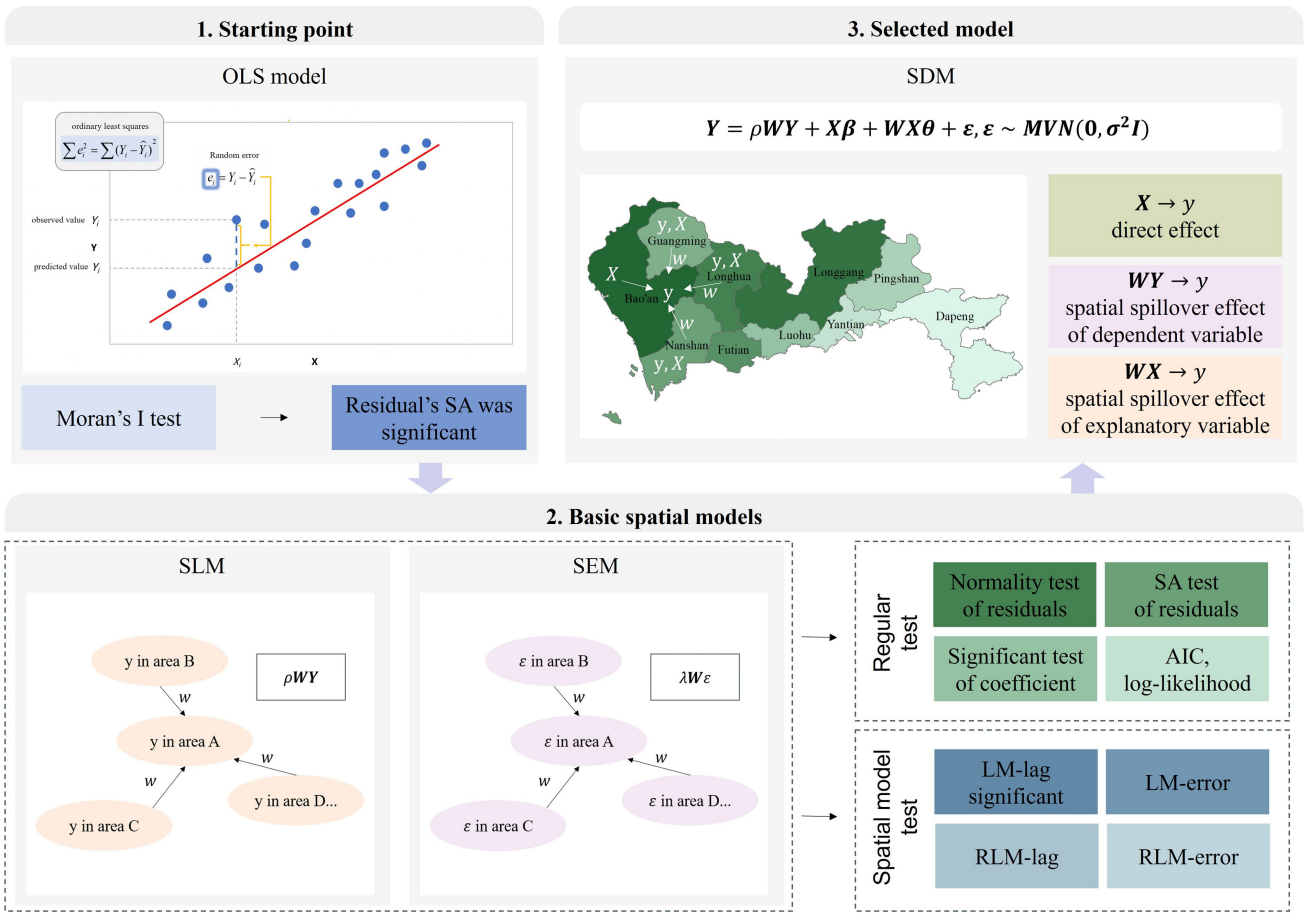
The model selecting procedure.

To validate the rationale for using the Spatial Durbin Model (SDM), we compared the statistical performance of multiple models: the multivariate linear regression model (OLS), spatial regression models (including the Spatial Lag Model (SLM) and Spatial Error Model (SEM)), and the SDM. Figure 3 outlines the model selection procedure used in this research, while Supplementary Tables A1, A2 provide the detailed results of model diagnostics.

**Figure 4:**
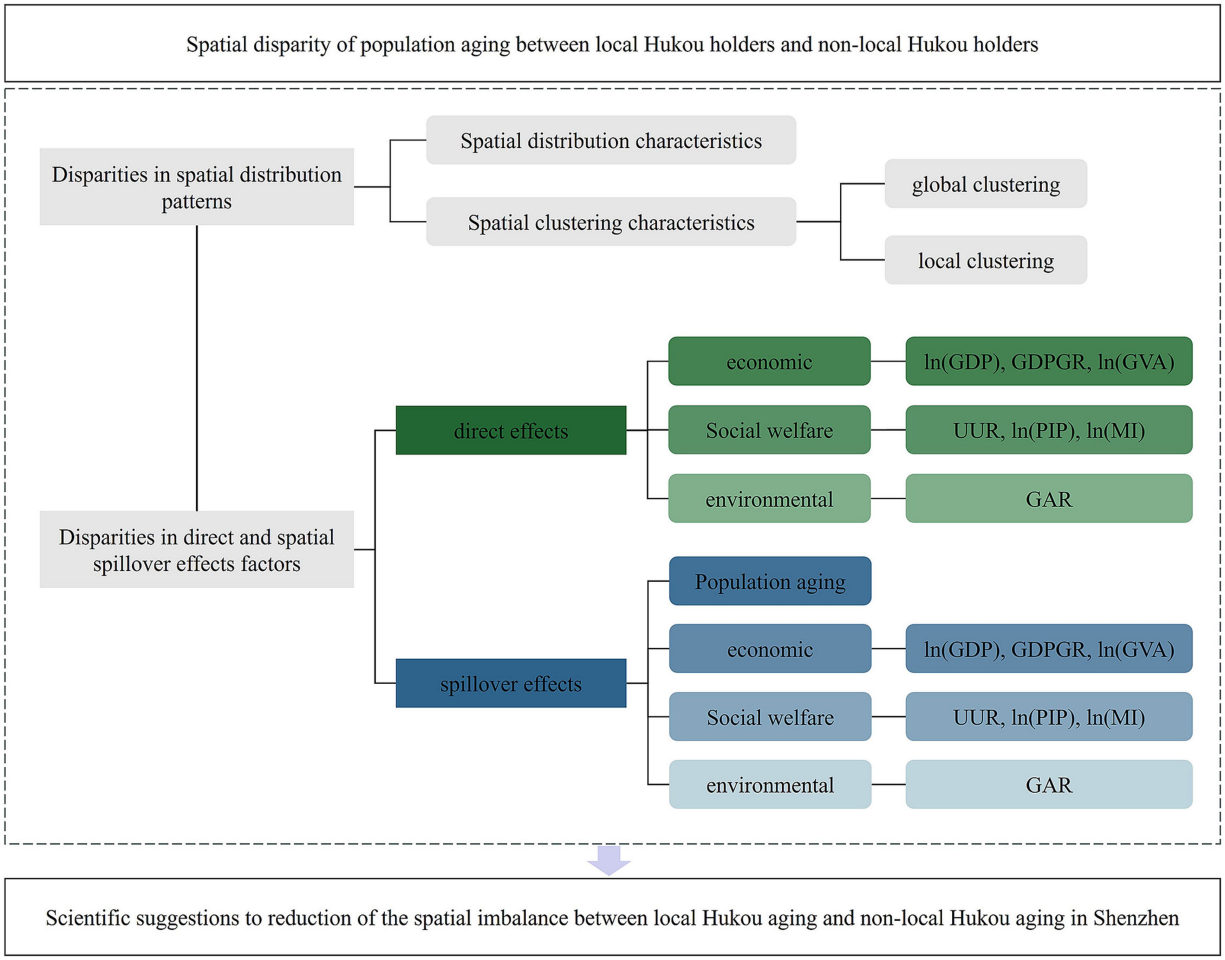
The logic of the research.

Figure 4 shows the overall logic of the research, which examines the spatial disparities in population aging among local and non-local Hukou individuals. The study focuses on spatial patterns and the distinct mechanisms through which various influential factors affect the two types of aging groups.

## 3 Spatial patterns of population aging of local Hukou holders and non-local Hukou holders in Shenzhen

### 3.1 Spatial distribution characteristics

**Figure 5:**
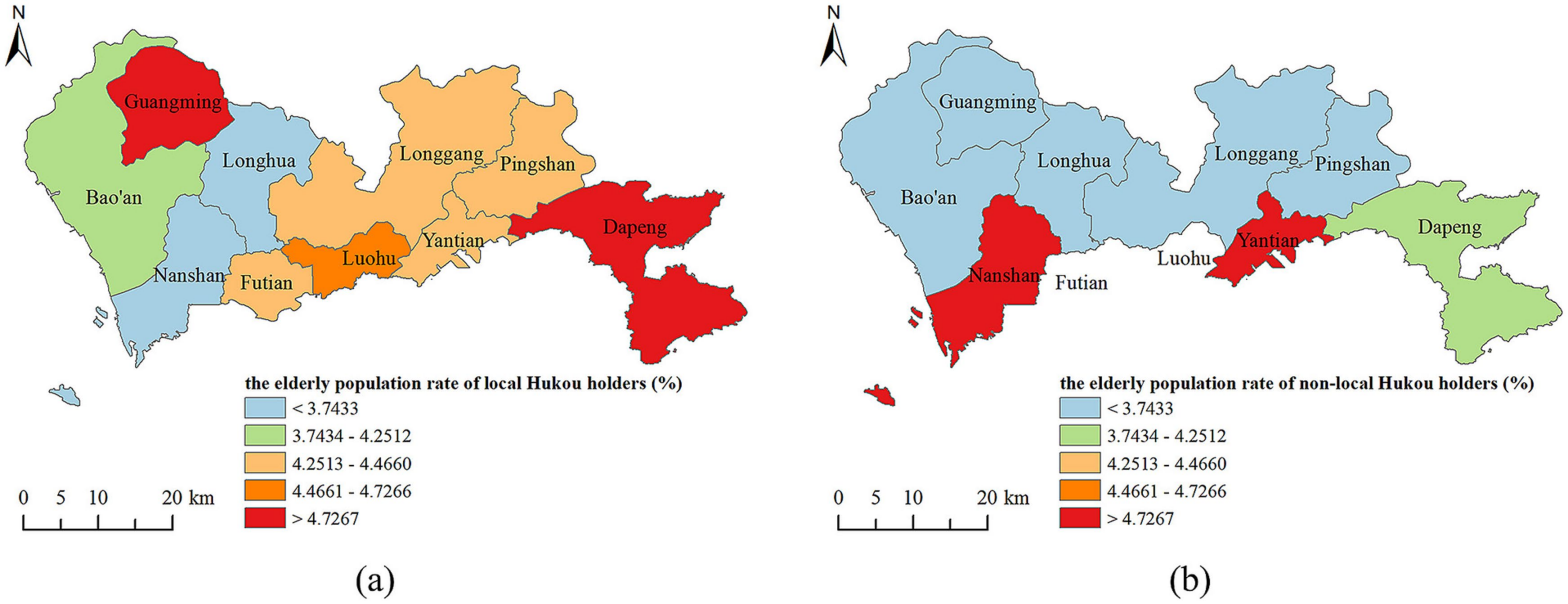
The spatial distributions of the older population rate of **(a)** local Hukou holders in 2020, and **(b)** non-local Hukou holders in 2020.

Figure 5 presents the spatial distribution maps of older population rates (aged 65 and above) for both local and non-local Hukou holders. Based on Jenks’ natural breaks classification method (36), this paper categorizes the older population rate of local Hukou holders in Shenzhen into five groups: (I) less than 3.743%, (II) 3.743–4.251%, (III) 4.251–4.466%, (IV) 4.466–4.726%, and (V) above 4.726%.

In 2020, the aging of the local Hukou population in Shenzhen showed regional variation (Figure 5a). Areas with low older populations, classified as Class I, are primarily located in western districts like Longhua and Nanshan. Regions with moderate older populations, classified as Class III, are mainly found in the central areas, including Luohu, Futian, Longgang, Pingshan, and Yantian districts. Regions with high older populations, classified as Class V, are dispersed in the northwestern (i.e., Guangming) and the southeastern (i.e., Dapeng) districts. Overall, the aging pattern of the local Hukou population in Shenzhen can be described as “rising from west to east.”

The distribution of aging rates among the non-local Hukou population presents a different pattern. As illustrated in Figure 5b, there are three levels of aging for the non-local Hukou population. The northern regions, which includes Baoshan, Guangming, Longhua, Longgang, and Pingshan districts, exhibit low aging rates and fall entirely within Class I. Dapeng district located in the southeast is categorized as Class II. The southern regions, comprising Nanshan, Futian, Luohu, and Yantian districts, display high aging rates and are classified entirely as Class V. To summarize, the non-local Hukou aging in Shenzhen presents the characteristics of “high in the south and low in the north.”

### 3.2 Spatial autocorrelation analysis of population aging in Shenzhen

Table 2 presents the global Moran’s I values for the older population rates of both local and non-local Hukou holders in 2020. The *p*-value for local Hukou aging rates is not significant, in contrast, the p-value for the aging rate of non-local Hukou holders is significant at the 0.1 level, with a Moran’s I value of 0.2531, suggesting a low positive spatial autocorrelation.

**Table 2:** Global Moran’s I of aging rate of local Hukou holders and non-local Hukou holders.

Aging rates	Moran’s I	E(I)	Z-score	*p*-value
Local aging rates	−0.0680	−0.1111	0.2115	0.36049
Non-local aging rates	0.2531	−0.1111	1.5771	0.06946

**Figure 6:**
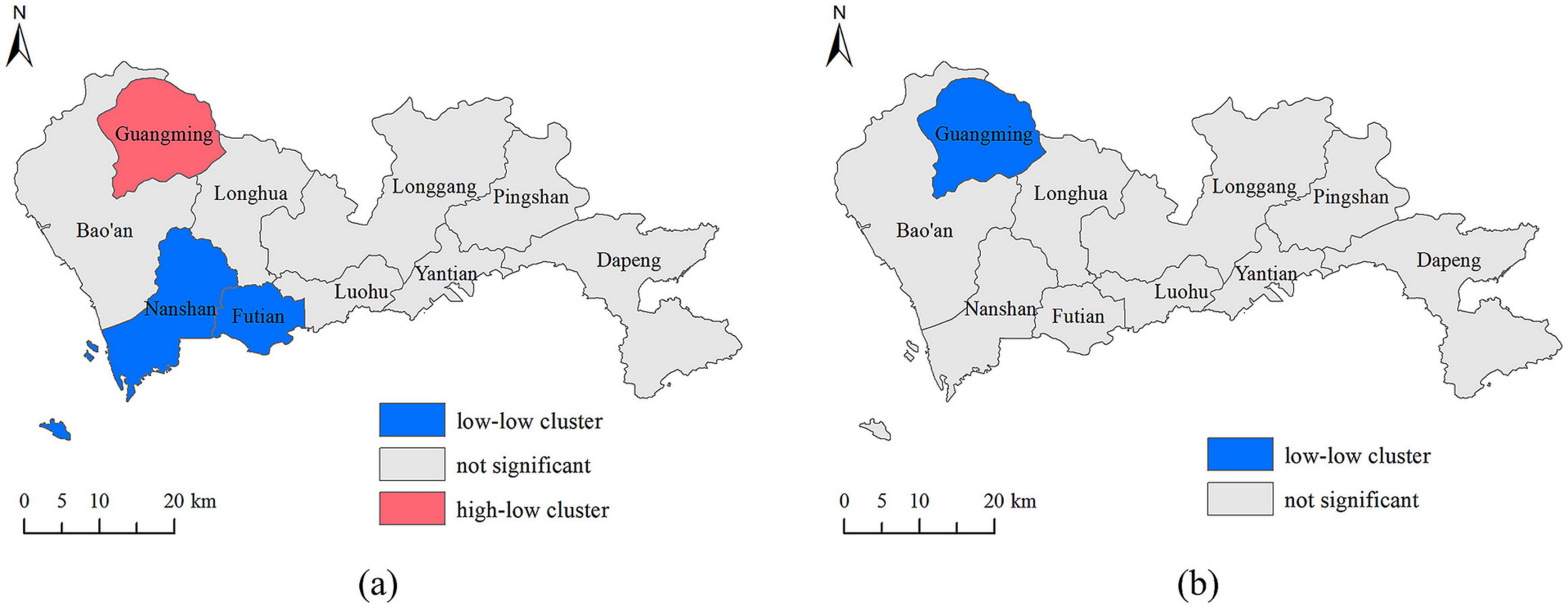
Local cluster maps of aging for **(a)** local Hukou holders, and for **(b)** non-local Hukou holders.

Figure 6 displays local cluster maps of aging rates for both local and non-local Hukou populations. For the local Hukou population, southwestern regions like Nanshan and Futian districts form a low-low cluster, while Guangming district in the northwest is part of a hight-low cluster. This indicates that low aging rates among the local Hukou population are concentrated around Nanshan and Futian districts, whereas Guangming district exhibits relatively high aging rates, surrounded by districts with low aging rates. For the non-local Hukou population, Guangming district in the northwest forms a low-low cluster, suggesting that both Guangming district and its neighboring districts have low aging rates. These local findings align with the discussions in subsection 3.1.

## 4 Spatial spillover effects of influential factors on two types of population aging

This section examines the effects of economic, social welfare, and environmental factors on the aging of both local and non-local Hukou holders in Shenzhen. Based on the results of the SDM model, the differences in spillover effects of influencing factors between local and non-local Hukou holders are analyzed and explained.

### 4.1 The opposite mechanism of impacting factors on the two types of aging

Table 3 summarizes some results of SDM, almost all of the explanatory variables significantly correlated with the two types of aging rates, both of which have strong positive spatial autocorrelation coefficients ρ , indicating the two types of aging rates have positive spatial spillover effects on their neighbors. It can be seen from Table 3 that almost all of the explanatory variables have opposite impacts on local Hukou aging rates and non-local Hukou aging rates.

**Table 3:** The estimated coefficients of SDM explanatory variables and related diagnosis.

Factor category/Statistics	Variables	Local aging rates		Non-local aging rates	
		Value	*p*-value	Value	*p*-value
Economic	(Intercept)	0.0014	7.09E-05	0.0024	0.0004
	ln(GDP)	0.0020	<2.2e-16	−0.0009	0.0000
	GDPGR	−6.7852	<2.2e-16	6.3731	0.0000
	ln(GVA)	−0.0004	0.0013	−0.0159	<2.2e-16
Social welfare	UUR	20.1250	<2.2e-16	−21.9540	<2.2e-16
	ln(PIP)	−0.0026	<2.2e-16	0.0156	<2.2e-16
	ln(MI)	0.0002	2.95E-09	−0.00008	0.2162
Environmental	GAR	0.0489	0.0301	−0.2292	0.0000
AIC	–	−22007.000	–	−20579.000	–
Log-likelihood	–	10739.9700	<2.22e-16	9257.9250	<2.22e-16
Wald statistic	–	17033.0000	<2.22e-16	56502.0000	<2.22e-16
Spatial autocorrelation parameter	–	0.9341	<2.22e-16	0.9695	<2.22e-16

#### 4.1.1 Economic indicators

Table 3 indicates that ln(GDP), GDP growth rate (GDPGR), and ln(GVA) are significant in both the local and non-local Hukou models. For local Hukou aging, ln(GDP) is positively correlated, whereas GDPGR and ln(GVA) are negatively correlated. In contrast, for non-local Hukou aging, GDPGR is positively correlated, while ln(GDP) and ln(GVA) are negatively correlated.

The observed correlation between economic factors and aging rates in Shenzhen can be explained by the city’s developmental history. In the early decades, central districts like Luohu and Futian amassed significant resources and achieved high GDP levels. As the earliest developed areas, they also experienced higher population densities. Over time, the initial settlers in these districts have aged, resulting in more pronounced aging among the local Hukou population in these high-GDP regions.

Beyond these established core areas, Shenzhen has been actively developing new districts to promote balanced growth and shared prosperity across the city. For instance, Guangming District has recently experienced high GDP growth rates due to its development as a scientific hub. In 2019, the Central Committee of the Communist Party of China and the State Council issued directives to support Shenzhen in becoming a pilot demonstration zone of socialism with Chinese characteristics, emphasizing its role in building a comprehensive national science center. The Shenzhen Municipal Government aims to transform Guangming District into an advanced science district by enhancing the innovation environment and attracting innovative talents. As technological and industrial development progresses in new districts like Guangming and Pingshan, they offer more high-quality jobs, drawing young migrants from other parts of Shenzhen and the conutry. Although these new areas have lower total GDP compared to early-developed districts, they are experiencing rapid GDP growth and thus attracting influx of young people which slows the aging process and reduces the local aging rate.

#### 4.1.2 Social welfare indicators

Table 3 reveals some interesting findings: UUR, ln(PIP), and ln(MI) are significant in local aging model, while ln(MI) is not significant in non-local aging model; additionally, the correlations between factors (UUR and ln(PIP)) and aging rates differ between the two models, showing opposite relationships.

The logarithm of the number of medical institutions, ln(MI) is positively correlated with local aging rates, suggesting that older individuals with local Hukou are more inclined to retire in areas offering superior medical care. However, for the non-local older population, medical care does not appear to be a primary consideration.

It is worth noting that regions with higher registered urban unemployment rates (UUR) tend to experience more pronounced aging among local Hukou holders and less pronounced aging among non-local Hukou holders. Additionally, areas with greater participation in pension insurance (ln(PIP)) exhibit lower aging rates for local Hukou holders and higher rates for non-local Hukou holders. This pattern aligns with Shenzhen’s migratory dynamics. A higher UUR indicates fewer job opportunities, prompting young people to leave the region and resulting in increased local aging. Conversely, greater participation in pension insurance suggests a robust workforce, leading to a lower local aging rate.

#### 4.1.3 Environmental indicator

The good air quality rate (GAR) is significantly positively correlated with aging in the local Hukou model and significantly negatively correlated in the non-local Hukou model. This suggests that regions with higher rates of good air quality experience more pronounced aging among the local Hukou population and less severe aging among the non-local Hukou population.

Economic growth and rising living standards increase material desires, prompting the older people to prioritize ideal retirement environments where ecological factors play a crucial role. Air pollution poses significant health risks to older adults (37), making good air quality a priority for them. In contrast, many non-local older individuals move to Shenzhen to accompany their working children, resulting in retirement choices that are often passive and less influenced by environmental or social welfare factors compared to those of local older residents.

In summary, with the exception of ln(GVA), all influential variables have opposite effects on the two types of aging. Compared to older adults with local Hukou holders, the aging population of non-local Hukou holders is more influenced by their children’s priorities. These children tend to prioritize economic development and job opportunities over social welfare and the natural environment of a region.

### 4.2 The opposite mechanism of impacting factors on the two types of aging in neighborhoods

Table 4 shows the coefficient values for the lag terms of SDM explanatory variables. The effects of W*ln(MI) and W*GAR on the aging of local Hukou holders are not significant, and the impact of W*ln(GDP) on the aging of non-local Hukou holders is also not significant. Besides GVA, other variables exhibit opposite spillover effects on the two types of aging in neighboring areas.

**Table 4:** The estimated coefficient values of the lag term of SDM explanatory variables.

Factor category	Lagged variables	Local aging rates		Non-local aging rates	
		Value	*p*-value	Value	*p*-value
Economic	W*ln (GDP)	−0.0020	< 2.2e-16	0.0002	0.2916
	W*GDPGR	4.8258	4.44E-16	−8.5295	8.28E-14
	W*ln (GVA)	0.0006	0.0003	0.0162	< 2.2e-16
Social welfare	W*UUR	−13.7750	2.22E-16	27.8960	< 2.2e-16
	W*ln (PIP)	0.0019	< 2.2e-16	−0.01585	< 2.2e-16
	W*ln(MI)	−0.00003	0.4766	0.0002	0.0029
Environmental	W*GAR	−0.0436	0.1735	0.1985	0.0010

#### 4.2.1 Economic indicators

All economic factors exhibit spillover effects on aging rates of local older population, but not all influence the aging rates of the non-local older population. Specifically, ln(GDP) has negative spillover effects on local aging rates, while the GDP growth rate (GDPGR) exerts positive spillover effects on the local aging rates. In contrast, ln(GVA) has positive spillover effects on the aging rates of both local and non-local Hukou populations.

The aging rate of non-local Hukou population is not sensitive to the ln(GDP) of neighboring areas, but is strongly correlated with the GDPGR of these regions. A 1% increase in GDPGR in surrounding areas results in an 8.5295% decrease in the aging rate of the non-local Hukou population. This suggests that older individuals without Shenzhen Hukou may relocate with their working children to the neighboring regions experiencing rapid economic development.

#### 4.2.2 Social welfare indicators

Almost all social welfare indicators show spillover effects on both types of aging rates except ln(MI) on local aging rate. The urban unemployment rate (UUR) negatively affects local Hukou aging but positively influences non-local Hukou aging. Conversely, ln(PIP) (pension insurance participants) positively impacts local Hukou aging and negatively affects non-local Hukou aging. Additionally, ln(MI) has positive spillover effects on non-local Hukou aging.

A 1% increase in UUR in surrounding areas increases the non-local Hukou aging rate by 27.896%, indicating a positive spillover effect. For example, if area A is near areas B, C, and D with high unemployment, workers and their older parents from these areas may move to area A for job opportunities, raising the non-local Hukou aging rate there. In contrast, a 1% increase in ln(PIP) in surrounding areas decreases the non-local Hukou aging rate by 0.01585%, suggesting that this population relocates with their working children to regions with more jobs. While ln(MI) does not correlate with non-local aging rates, it shows positive spillover effects on this aging type.

#### 4.2.3 Environmental indicator

Interestingly, the good air quality rate (GAR) is positively correlated with local aging rates but does not exhibit spillover effects on this type of aging. Conversely, while GAR does not significantly correlate with non-local aging rates, it does show positive spillover effects on this group. Table 4 indicates that the aging rate of the non-local Hukou population is positively influenced by the spillover effects of both the medical environment and air quality rate.

**Figure 7:**
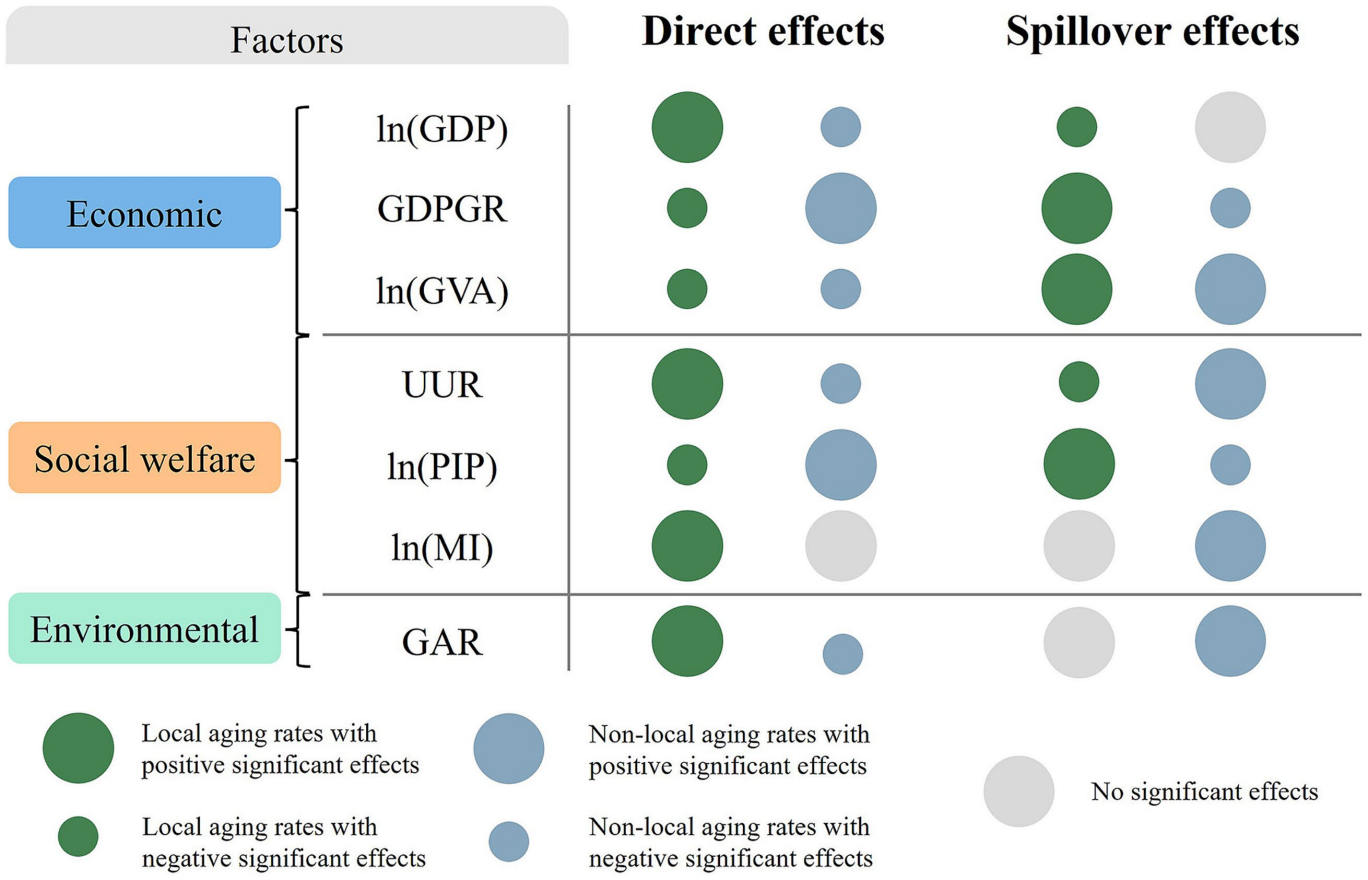
The influencing mechanisms of factors on local aging rates and non-local aging rates.

Figure 7 outlines how economic, social welfare, and environmental factors affect local and non-local aging rates, it can be seen that most variables have opposite effects on the two aging types. For instance, GDPGR negatively affects local aging rates but positively impacts non-local aging rates, while it has positive spillover effects on local aging rates and negative effects on non-local rates. The ln(MI) shows no direct impact on non-local aging rates but has positive spillover effects. Both ln(MI) and good air quality rates (GAR) positively influence local aging rates directly, but neither has spillover effects. The results implicate that non-local aging population tends to live in areas with higher GDP growth and more job opportunities, as well as near regions with superior medical and natural environments favored by local older individuals.

## 5 Discussion

As China’s youngest major city and primary migrant receiving metropolis, Shenzhen faces impending compressed aging despite maintaining an overall aging rate below the international threshold for an aging society. This phenomenon stems from dual pressures: (i) the simultaneous retirement of pioneering migrant laborers (post-1980 “Shenzhen Speed” builder), and (ii) the continuously influx of accompanying older adults migrating with younger generations. Critically, most accompanying older adults lack Shenzhen Hukou and possess limited pathways to acquire it^2^. Through a novel perspective of the Hukou system (i.e., the household registration system), this research employs spatial autocorrelation analysis and the spatial Durbin model (SDM) to reveal distinct spatial clustering patterns among older populations by Hukou status and differential impacting mechanisms of economic, social welfare, and environmental factors. The findings provide evidence for targeted policy responses to Shenzhen’s unique compressed aging.

While the Hukou perspective has been applied in numerous studies on aging (38–41), few have examined population aging under compressed aging transitions in major migrant receiving cities. Conversely, spatial autocorrelation analysis and the spatial Durbin model (SDM) have been widely used in aging studies (42–46), and the seven influencing variables across three categories are empirically validated in existing literature (8, 9, 15–19). Building on this foundation, this section examines the influences of demographic factors on Shenzhen’s aging and derives policy implications from our analytical findings.

### 5.1 Demographic factors on Shenzhen’s aging

Population aging occurs when the age distribution of a population shifts towards older ages, typically indicated by an increase in the number of older individuals and a rise in the median age (47). Key demographic factors influencing aging include birth rate, death rate, and migration (48). Lower birth rates and a smaller proportion of young children, and more older individuals accelerate aging (48). Migration alters the age structure; an influx of young people can offset the impact of an increasing older population, mitigating regional aging, while an outflow of young people intensifies it (49). Additionally, indirect factors such as the economy, environment, and education level influence aging by affecting direct factors like birth and migration rates (50, 51).

Due to incomplete datasets on population-related indicators in Shenzhen’s districts, this paper focuses on quantitatively analyzing the effects of indirect factors—economy, social welfare, and environmental factors—on aging. We then discuss how these indirect factors influence direct determinants of population aging, such as birth rate, death rate, and migration rate.

Researches indicate that healthcare (52), economic development (53) and national politics (54) affect birth rates. Despite Shenzhen’s strong medical and economic conditions, its birth rate remains low. This is partly because improved healthcare reduces death rates, extending lifespan and increasing population aging. As Chinese society rapidly develops and living standards rise, economically advanced areas often experience lower fertility levels (55). Although the government has recently relaxed fertility policies, the impact on increasing Shenzhen’s birth rate is limited.

Researches also indicate that death rates are influenced by social welfare (56), economic development (57), and air quality (58). Shenzhen’s low death rate is attributed to its strong economic development and robust urban infrastructure. Additionally, enhancements in medical care, health services, and a more comprehensive social security system significantly contribute to reducing mortality rates. Poor air quality is linked to higher mortality rates, as it increases the risk of chronic obstructive pulmonary disease, cardiovascular disease, and cerebrovascular disease, while also impairing lung and immune functions.

The economy significantly influences population migration (59, 60). Urban in-migration rates are positively linked to local income levels, technological advancement, and cultural development (61). Due to its rapid economic growth, labor-intensive industries, and high demand for workers, Shenzhen attracts many young people from other provinces, benefiting from the ‘demographic dividend.’ The influx of young people outweighs the increase in the older population, resulting in Shenzhen’s aging rate being significantly lower than the national average. Additionally, the quality of urban public services affects migrants’ willingness to settle; higher service levels increase this willingness (62). Environmental factors also play a role in migration, as economic development heightens the desire for a better quality of life, leading people to prefer areas with a good environment and economic vitality. Older individuals, particularly sensitive to air pollution’s health effects, seek retirement in areas with better air quality (37).

### 5.2 Implications for coping with uneven development of local Hukou aging and non-local Hukou aging in Shenzhen

Despite maintaining its status as China’s youngest metropolis, Shenzhen confronts a paradoxical demographic crisis characterized by accelerated population aging. As the nation’s typical migrant receiving city, it faces a unique “compressed aging” issue – a dual pressure stemming from the concurrent retirement of pioneering migrant workers (post-1980 “Shenzhen Speed” builder) and increasing older dependents accompanying younger migrants. Unlike the gradual aging transitions observed in other cities in China, this clustered aging transition creates unprecedented governance challenges in social sustainability.

Through a Hukou-differentiated analytical framework, this study investigates the spatial disparities between native-registered and migrant older population. Our policy recommendations address three aspects: healthcare resource allocation, psycho-social infrastructure, and Hukou policy.

#### 5.2.1 Promote the formation of a diversified supply pattern of care services for older population

As society ages further, the varying pension needs among different groups will increase, necessitating a more diversified pension model. It’s crucial to develop care services encompassing social welfare, healthcare, cultural activities, and other relevant areas. In southern districts like Nanshan, Futian, Luohu, and Yantian, where non-local Hukou aging is more concentrated, efforts should focus on facilitating cross-regional medical insurance settlements to enable real-time processing, reducing procedures and waiting times, and establishing expedited treatment pathways. Additionally, there should be an expansion of remote care facilities and public services for the non-local Hukou aging population. Regional green space planning should also be enhanced, incorporating features like rest areas and health walks to accommodate the needs of the older adults.

#### 5.2.2 Create a community environment that facilitates communication for the non-local Hukou older individuals

The social circle of older residents with non-local Hukou is relatively limited, resulting in fewer social activities compared to older adults with local Hukou. Therefore, in southern districts like Nanshan, Futian, Luohu, and Yantian, where non-local Hukou aging is more concentrated, it is important to enhance public activity spaces to facilitate interaction and integration between these groups. This can be achieved by investing in multifunctional community centers that offer leisure, entertainment, learning, and socializing opportunities, equipped with older-friendly facilities such as reading areas, chess and card rooms, tea rooms, and health lecture spaces. Additionally, organizing regular cultural festivals and performances can encourage participation from both groups, fostering mutual understanding through cultural exhibitions and exchanges.

#### 5.2.3 Optimize Hukou point policy and extend the public services from registered population to resident population

The current points-based Hukou acquisition policy in Shenzhen disproportionately emphasizes economic contributions through metrics such as property ownership, social security payments, and educational credentials. We propose incorporating “non-local care support” into the scoring matrix to recognize applicants’ familial responsibilities. This addition would not only address the policy’s current oversight of familial obligations but also incentivize family-unit migration patterns. By reducing seasonal migratory population fluctuations and stabilizing permanent residency, urban planners could optimize public service allocation with greater demographic predictability.

Furthermore, implementing extension of the public services from registered older population to residential older population would enhance both equity in service accessibility and operational efficiency of public resources. Such reform directly aligns with the strategic objectives outlines in Shenzhen’s 14th Five-Year Plan for Population and Social Development (2021–2025) (63) particularly regarding the optimization of population structure and social service delivery systems.

Although Shenzhen has not yet met the threshold for an aging society, our findings provide actionable preparations for its impending demographic transition. Furthermore, as a typical immigrant city, Shenzhen’s policy recommendations can provide insights for other fast-growing cities with similar migration-driven population structures.

## 6 Conclusion

As China’s youngest metropolis, Shenzhen paradoxically faces rapid and compressed population aging. Although its average aging (population aged 65+) rate has not yet exceeded the 7% international threshold for an aging society, urgent preparedness is required given its unique status as a migrant-dominated megacity with unparalleled demographic dynamics in China. To address this challenge, this study adopts the Hukou system perspective to compare the spatial distribution of older populations between local and non-local Hukou holders, and investigate the economic, social welfare, and environmental factors affecting aging in these groups. Our conclusions are as follows:

1. The spatial distribution of aging differs between local and non-local Hukou holders. Local Hukou aging trends increase from west to east, while non-local Hukou aging is higher in the south and lower in the north.
2. Globally, the non-local Hukou older population shows a low degree of positive spatial autocorrelation. Locally, local Hukou aging rates show low-low clustering in southwestern districts like Futian and Nanshan, and high-low clustering in a northwestern (i.e., Guangming) district. In contrast, non-local Hukou aging rates exhibit low-low clustering in Guangming district, indicating a low-value area centered there.
3. Shenzhen’s population aging is significantly linked to economic, social welfare, and environmental factors. Older residents with local Hukou prefer high-quality environments like good health care and air quality, while older adults with non-local are often dominated by their working children who value more on economic factors and work opportunities.
4. To prevent regional imbalances in aging between local and non-local Hukou populations, it is beneficial to promote diverse care services and create community environments that enhance communication for non-local Hukou aging population. In addition, incorporating “non-local care support” into the current Hukou point policy and extending the public services from registered population to resident population are also helpful for building a sustainable and aging-friendly society.

Through the institutional lens of China’s Hukou system, this study reveals that Shenzhen’s registered and migrant older populations exhibit divergent spatial configurations and determinant mechanisms. Our findings necessitate targeted policy interventions across three domains: (1) efficiency-aware healthcare resource allocation, (2) culturally responsive psycho-social infrastructure development, and (3) Hukou point system reforms incorporating care obligations for older adults. This institutional analysis provides a transferable framework for migrant receiving cities undergoing accelerated urbanization – particularly those confronting compassed aging transitions under similar institutional constraints.

This study has several limitations. First, the analysis relies exclusively on cross-sectional data from 2020, which constrains our ability to examine temporal dynamics. Future research should incorporate longitudinal datasets to trace the evolving relationships between aging rates and their determinants. Second, while our SDM-derived estimates of direct and spillover effects provide empirical foundations for causal inference, this study does not establish causal relationships. Investigating causal pathways between aging rates and these factors represents a critical frontier, constituting a clear priority for our future research.

## Data Availability

The raw data supporting the conclusions of this article will be made available by the authors, without undue reservation.

## Footnotes

^1^ https://tjj.sz.gov.cn/zwgk/zfxxgkml/tjsj/tjnj/content/post_10688160.html

^2^A fundamental eligibility criterion for local Hukou application is age compliance, as exemplified by Shenzhen requirements: male applicants must be less than 55 years old; and female applicants must be less than 50 years old (official source: https://ga.sz.gov.cn/gkmlpt/content/10/10660/post_10660458.html#842). Our study focuses specifically on the proportion of residents aged over 65 years old.
