# Supplementary material for: Spatial disparities of population aging in Shenzhen: from China’s Hukou perspective
Source: Front Public Health. 2025 Aug 4;13:1614007. doi: 10.3389/fpubh.2025.1614007 (PMC12358437; doi:10.3389/fpubh.2025.1614007)
Supplement: Supplementary file 1 [file Table_1.DOCX]

Supplementary Material

# Supplementary Tables

**The details of rationale to build a spatial Durbin model**

Table A1 shows that the Moran's I values for the OLS model residuals exceed 0.7, indicating significant spatial autocorrelation and violating the OLS model's assumption of error term independence. Therefore, spatial regression models are more appropriate in this context.

**Table A1** Results of model diagnosis of the OLS

| **Statistics** | **local Hukou holders** | | **non-local Hukou holders** | |
| --- | --- | --- | --- | --- |
|  | **value** | **P-value** | **value** | **P-value** |
| **AIC** | -17676.77 | —— | -14070.83 | —— |
| **Moran’I test statistic for residuals** | 0.7239 | < 2.22e-16 | 0.784 | < 2.22e-16 |
| **R^2^** | 0.6698 | —— | 0.6292 | —— |

Table A2 presents the model fitting and diagnostic results for the Spatial Lag Model (SLM) and Spatial Error Model (SEM). The spatial autocorrelation parameters for the SLM are 0.8378 for local Hukou holders and 0.8374 for non-local Hukou holders, indicating positive spillover effects where the aging rate in one area is influenced by neighboring areas. The SEM parameters are 0.9625 and 0.9745 for local and non-local Hukou populations, respectively. Except for the SLM of local Hukou holders, the Moran’s I tests for other model residuals indicate significant but minimal autocorrelation, suggesting these models effectively capture the spatial dependencies in the data.

**Table A2** Results of model diagnosis of the SLM and SEM

| **Statistics** | **local Hukou holders** | | | | **non-local Hukou holders** | | | |
| --- | --- | --- | --- | --- | --- | --- | --- | --- |
|  | **SLM** | | **SEM** | | **SLM** | | **SEM** | |
|  | **value** | **P-value** | **value** | **P-value** | **value** | **P-value** | **value** | **P-value** |
| **Spatial autocorrelation parameter** | 0.8378 | < 2.22e-16 | 0.9625 | < 2.22e-16 | 0.8374 | < 2.22e-16 | 0.9745 | < 2.22e-16 |
| **AIC** | -20664.64 | —— | -21332.98 | —— | -20664.64 | —— | -18417.00 | —— |
| **Log-likelihood** | 10342.32 | —— | 10676.49 | —— | 8385.227 | —— | 10286.1400 | —— |
| **Moran’I test statistic for residuals** | 0.1807 | 0.00001 | -0.0458 | 0.00002 | 0.0592 | 0.00001 | -0.0476 | 0.00003 |
| **R^2^** | 0.9052 | —— | 0.9595 | —— | 0.9363 | —— | 0.9608 | —— |
| **LM (lag)** | 2178.6426 | < 2.22e-16 | —— | —— | 2451.1558 | < 2.22e-16 | —— | —— |
| **LM (error)** | —— | —— | 2759.8612 | < 2.22e-16 | —— | —— | 2604.9825 | < 2.22e-16 |
| **Robust LM (lag)** | 12.9996 | 0.00031 | —— | —— | 65.0115 | < 2.22e-16 | —— | —— |
| **Robust LM (error)** | —— | —— | 594.2183 | < 2.22e-16 | —— | —— | 218.8382 | < 2.22e-16 |

The R^2^ and AIC values for both SLM and SEM indicate a good fit. To refine model selection, we used the Lagrange Multiplier (LM) and Robust Lagrange Multiplier (RLM) tests. As shown in Table A2, the LM tests for both SLM and SEM are significant, and the RLM test for SEM is also significant, suggesting consideration of the Spatial Durbin Model (SDM). Table 3 presents the SDM diagnostic results. The likelihood ratio (LR) and Wald tests, with p-values below 0.01, reject the null hypothesis that the SDM can be simplified to SLM or SEM, confirming the SDM's suitability. Additionally, the SDM demonstrates superior AIC and log-likelihood values compared to SLM and SEM.
